# Supplementary figures and images for: Functional regression clustering with multiple functional gene expressions
Source: PLoS One. 2024 Nov 25;19(11):e0310991. doi: 10.1371/journal.pone.0310991 (PMC11588248; doi:10.1371/journal.pone.0310991)

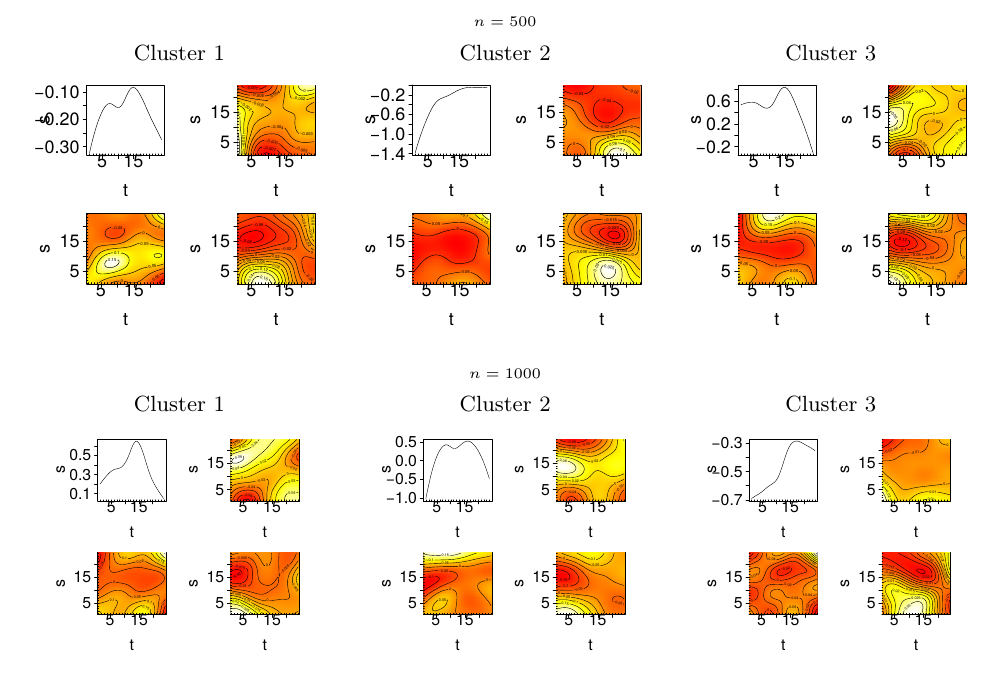

Supplement: S1 Fig — (TIF) [file pone.0310991.s001.tif]

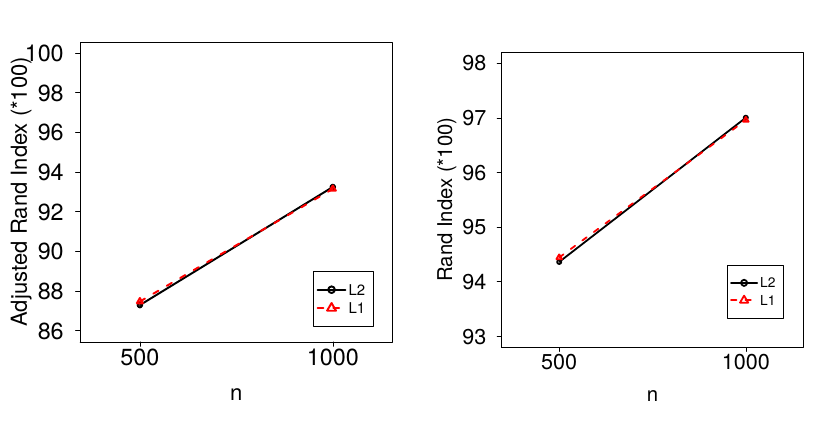

Supplement: S2 Fig — (TIF) [file pone.0310991.s002.tif]

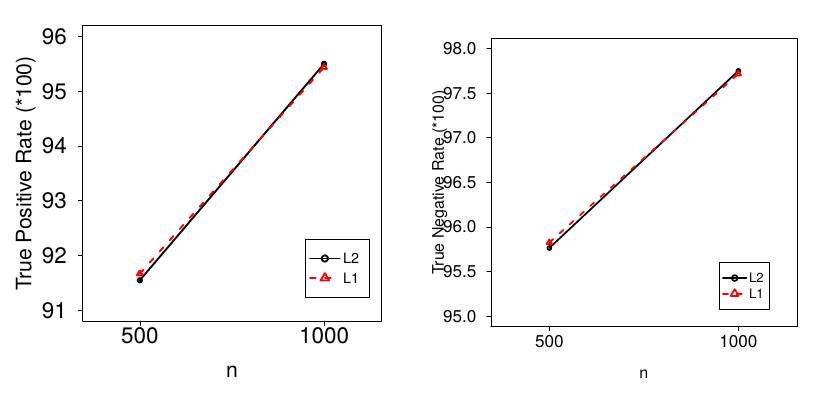

Supplement: S3 Fig — n = 500, 1000. (TIF) [file pone.0310991.s003.tif]

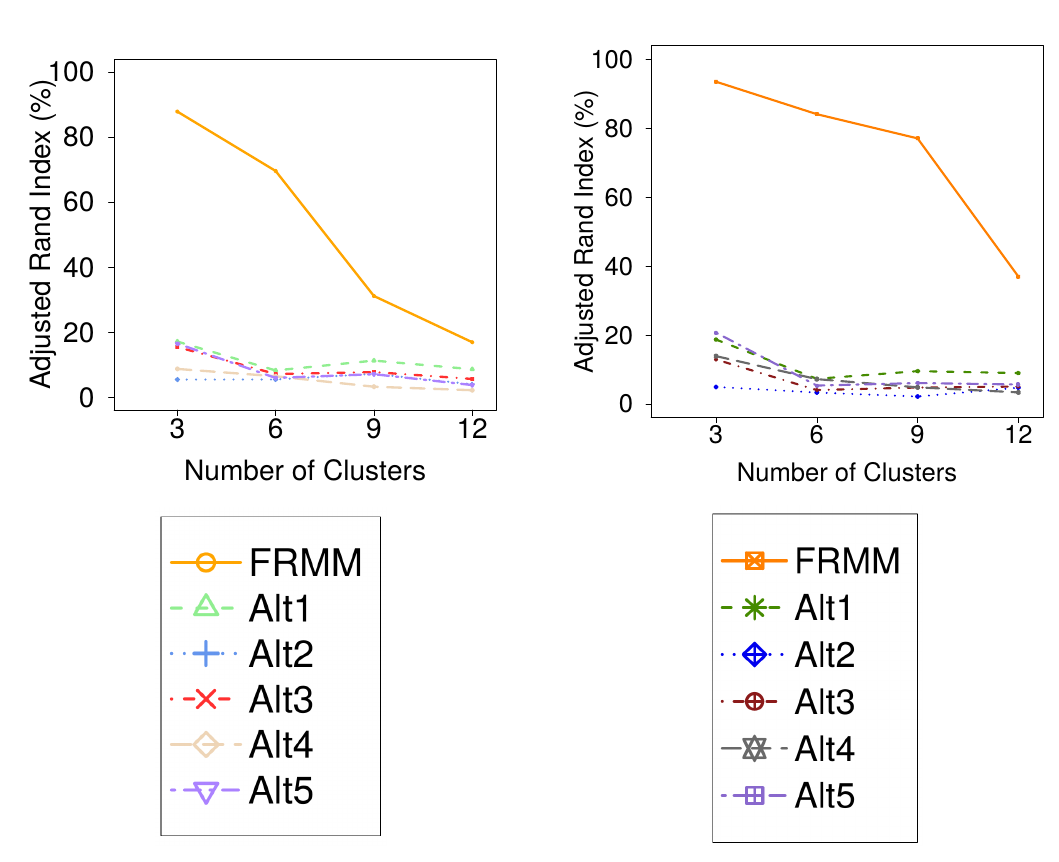

Supplement: S4 Fig — n = 500, 1000. (TIF) [file pone.0310991.s004.tif]

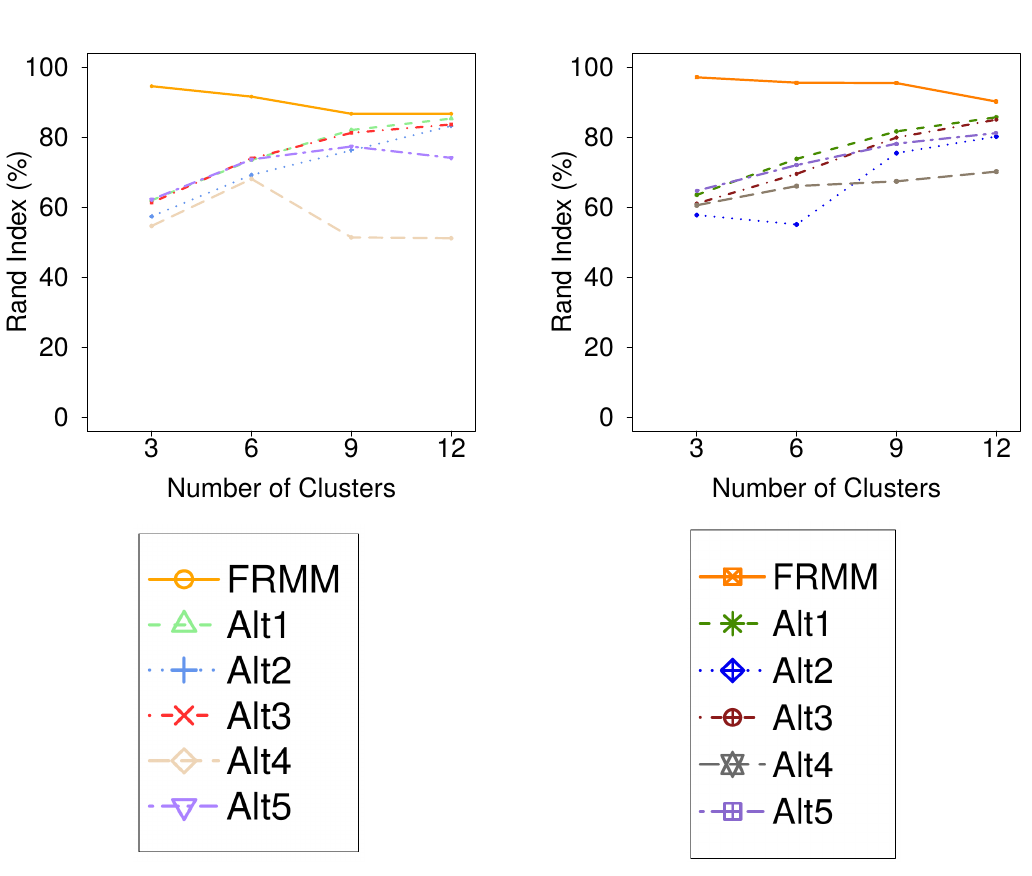

Supplement: S5 Fig — n = 500, 1000. (TIF) [file pone.0310991.s005.tif]

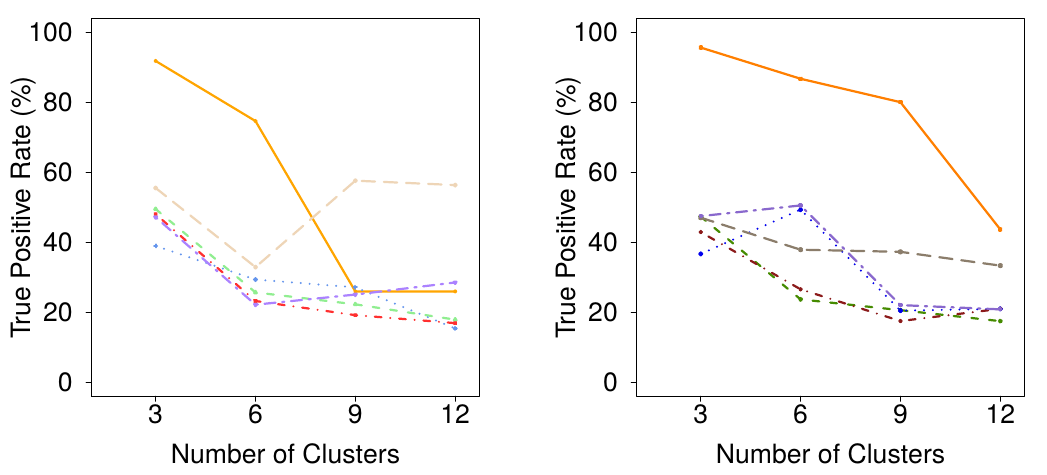

Supplement: S6 Fig — n = 500, 1000. (TIF) [file pone.0310991.s006.tif]

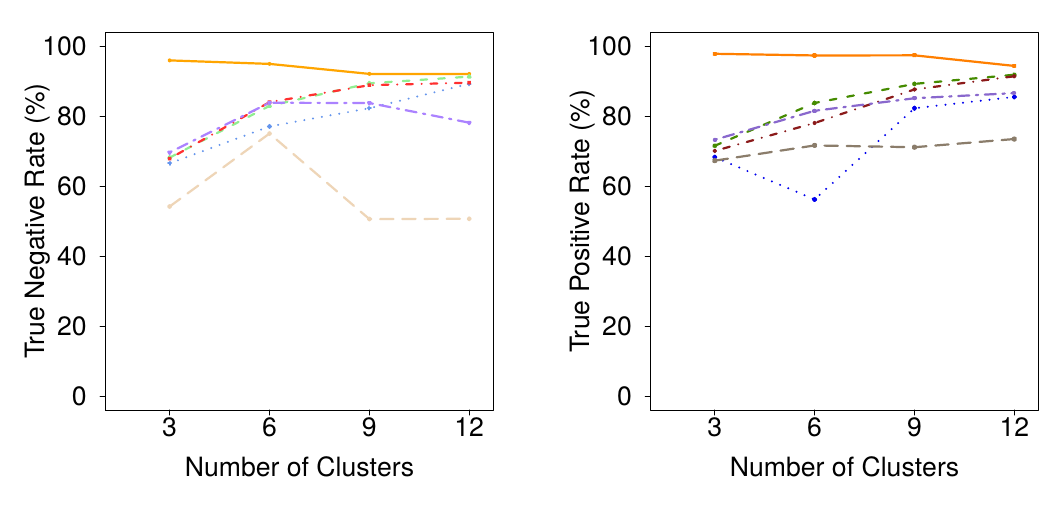

Supplement: S7 Fig — n = 500, 1000. (TIF) [file pone.0310991.s007.tif]

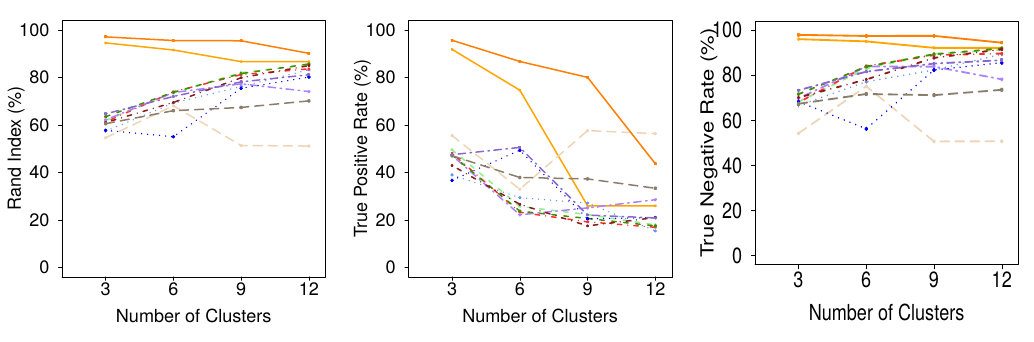

Supplement: S8 Fig — (TIF) [file pone.0310991.s008.tif]

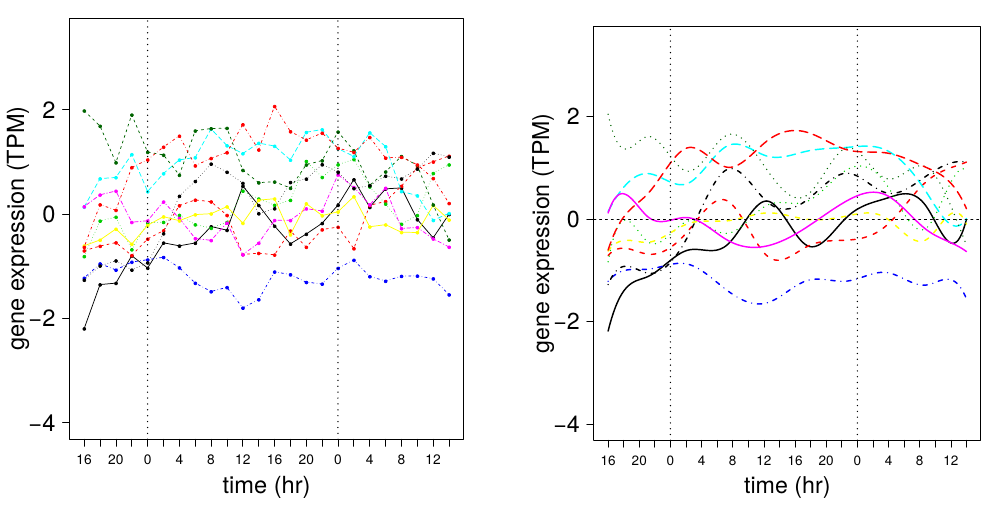

Supplement: S9 Fig — (TIF) [file pone.0310991.s009.tif]
