# Supplementary material for: Functional regression clustering with multiple functional gene expressions
Source: PLoS One. 2024 Nov 25;19(11):e0310991. doi: 10.1371/journal.pone.0310991 (PMC11588248; doi:10.1371/journal.pone.0310991)
Supplement: Attachment — Submitted filename: Peer Review for Functional Regression paper.pdf [file pone.0310991.s018.pdf]

Peer Review for "Functional Regression Clustering with Multiple Functional Gene Expressions"

This manuscript presents a novel method for clustering gene expression data using a function-on-function regression model. The proposed method aims to identify clusters of genes with similar changes in their expression profiles under different experimental conditions, providing useful biological insights. The manuscript includes a detailed methodology, validation through simulations, and an application to real gene expression data.

It is a well-written manuscript with several strengths. Firstly, the manuscript introduces a novel clustering method that leverages functional regression models, allowing for multiple functional explanatory variables. This approach is innovative and addresses a gap in the current methods for clustering gene expression data. Secondly, the authors validate their method through extensive simulations, demonstrating its effectiveness compared to existing methods. This thorough validation adds significant credibility to their approach. Lastly, the application of the method to gene expression data reveals clusters enriched for biologically meaningful functions, highlighting the potential of the method to uncover novel biological insights.

Nevertheless, it could be better if the authors make any improvements with clarity in method description, comparison with alternative methods, and about real data application. While the methodology is described in detail, some sections could benefit from additional clarity and simplification to make the complex statistical methods more accessible to a broader audience. The manuscript compares the proposed method with several existing clustering methods. However, more detailed discussions on why certain methods perform better or worse in specific scenarios would be beneficial. The application to real gene expression data is compelling, but additional context on the biological implications of the findings could strengthen this section.

Here are three questions for discussion.

1) Parameter Selection: How sensitive is the proposed method to the selection of parameters such as the number of clusters (K) and the choice of functional principal components? Can the authors provide guidelines or criteria for optimal parameter selection in practical applications?

2) Computational Efficiency: Given the iterative nature of the proposed algorithm and the use of consensus clustering, what are the computational demands of the method? How

does it scale with increasing data size, and are there any strategies to improve its efficiency?

3) Biological Validation: The manuscript demonstrates that the identified clusters are enriched for genes with similar biological functions. Can the authors provide examples of specific biological pathways or processes that were identified using their method and discuss their significance?
