# Supplementary material for: Functional regression clustering with multiple functional gene expressions
Source: PLoS One. 2024 Nov 25;19(11):e0310991. doi: 10.1371/journal.pone.0310991 (PMC11588248; doi:10.1371/journal.pone.0310991)
Supplement: Attachment — Submitted filename: revisionResponse.docx [file pone.0310991.s019.docx]

PONE-D-24-08404

Functional regression clustering with multiple functional gene expressions

PLOS ONE

Journal Requirements:

When submitting your revision, we need you to address these additional requirements.

0.1 Please ensure that your manuscript meets PLOS ONE's style requirements, including those for file naming. The PLOS ONE style templates can be found at
<https://journals.plos.org/plosone/s/file?id=wjVg/PLOSOne_formatting_sample_main_body.pdf> and
<https://journals.plos.org/plosone/s/file?id=ba62/PLOSOne_formatting_sample_title_authors_affiliations.pdf>

**This has been completed**

0.2 Please expand the acronym “EPSRC and BBSRC” (as indicated in your financial disclosure) so that it states the name of your funders in full.
This information should be included in your cover letter; we will change the online submission form on your behalf.

-EPSRC and BBSRC are now named in full in the revised cover letter. Thank you for changing the submission form on our behalf. We have removed the funding information from the acknowledgement section of the manuscript as specified in 0.3.

0.3 Thank you for stating the following in the Acknowledgments Section of your manuscript: "This project was funded by the Alan Turing Institute Research Fellowship under EPSRC Research grant (TU/A/000017) to DE; SRC/BBSRC Innovation Fellowship (EP/S001360/1) to DE and SC. ST would like o thank the Isaac Newton Institute for Mathematical Sciences, Cambridge, for support
and hospitality during the programme Statistical Scalability where work on this paper
was undertaken. This work was supported by EPSRC grant no EP/R014604/1."
We note that you have provided funding information that is not currently declared in your Funding Statement. However, funding information should not appear in the Acknowledgments section or other areas of your manuscript. We will only publish funding information present in the Funding Statement section of the online submission form.
Please remove any funding-related text from the manuscript and let us know how you would like to update your Funding Statement. Currently, your Funding Statement reads as follows: "This project was funded by the Alan Turing Institute Research Fellowship under EPSRC Research grant (TU/A/000017) to DE;
EPSRC/BBSRC Innovation Fellowship (EP/S001360/1) to DE and SC. ST would like to thank the Isaac Newton Institute for Mathematical Sciences, Cambridge, for support and hospitality during the programme Statistical Scalability where work on this paper was undertaken. This work was supported by EPSRC grant no EP/R014604/1.

Engineering and Physical Sciences Research Council (EPSRC): <https://www.ukri.org/councils/epsrc/>
Alan Turing Institute: <https://www.turing.ac.uk/>
Biotechnology and Biological Sciences Research Council (BBSRC): <https://www.ukri.org/councils/bbsrc/>
Isaac Newton Institute for Mathematical Sciences: <https://www.newton.ac.uk/>

The funders did not play any role in the study design, data collection and analysis, decision to publish or preparation of the manuscript."

Please include your amended statements within your cover letter; we will change the online submission form on your behalf.

**We now include our amended funding statements in the cover letter and have removed the funding information from the acknowledgements section.**

0.4 We notice that your supplementary figures are uploaded with the file type 'Figure'. Please amend the file type to 'Supporting Information'. Please ensure that each Supporting Information file has a legend listed in the manuscript after the references list.

**We now upload the supplementary figures under the file type supporting information.**

**We ensure that each SI file has a legend listed in the manuscript after the reference list.**

Additional Editor Comments:

0.5 The authors please be aware of an inconsistency regarding the corresponding author's name. In the submission process, the corresponding author is identified as Daphne Ezer, but elsewhere, including in the cover letter and the manuscript, the corresponding author is identified as Susana Conde. Please help straighten this out.

**This has been straightened out. Ezer is the corresponding author and Conde is the first author. This is consistent with our roles in the project, as Conde led the implementation and testing of the solution and Ezer developed the core algorithm and led the project management. Ezer also has a more consistent e-mail address for addressing queries than Conde.**

0.6 I tried to access the R code mentioned in the manuscript at <https://gitlab.com/sconde778/frmm_rpackagefunctions.git>. However, I was prompted to create a personal account and then encountered a 404 page. If possible, please save your R code in an easily accessible web repository, such as GitHub. Ensure your code sharing is functional to facilitate the review process moving forward smoothly.

**We are sorry for the error: it appears that we forgot to change the privacy settings in the Gitlab repository. However, now we have ported the whole project to Github, so that it is part of the Ezer Lab main repository, giving it more visibility. It is now available on:** [**https://github.com/stressedplants/FRMM**](https://github.com/stressedplants/FRMM)

Please also be aware that one of the reviewer's comments is provided in an attachment. If you cannot see it, please contact PLOS ONE for assistance.

***************************************
Reviewers' comments:

Reviewer's Responses to Questions

**Comments to the Author**

Reviewer #1: Review of “Functional regression clustering with multiple functional gene expressions”

Summary

This manuscript presents a novel clustering method, functional regression clustering (FRECL), for analyzing temporal gene expression profiles. FRECL utilizes a concurrent functional linear regression model and is inspired by the traditional K-means clustering algorithm. Focusing on the associations between response and explanatory variables, the method updates each gene’s cluster label based on the best-fitted functional regression model with the smallest residual norm, aiming to identify groups of differentially expressed genes linked to biological processes over time. By treating temporal gene expression observations as functions, FRECL effectively groups genes with varying temporal expression profiles under different conditions while capturing similar changes across treatments. This approach provides a new tool for understanding gene expression dynamics and their biological implications. The authors did a good job investigating algorithm properties, i.e., the effect of the number of clusters, sample size, sensitivity study, convergence, etc. However, I hope some major and minor concerns can be addressed.


Major comments

1.1	In Line 89, the manuscript discusses the extension to settings with distinct domains for the response and each predictor. It would be better if the discussion section re-commented on this possible extension to different domains.

**Indeed line 89 states: ‘While we assume that the functional responses and predictors are all defined on the same domain T , our methods can easily be extended to settings with distinct domains for the response and each predictors’ We agree that it would be useful to revisit this statement in the discussion section.**

**We have added an additional statement on line 487: “There are several avenues by which FRECL could be extended in the future. For instance, our current implementation assumes that the response and predictors have the same domain. However, it will be possible to extend the method by first scaling the time domains, an extension that would not fundamentally alter the algorithm.”**

1.2 In Line 105, equation (4). It would be clearer if math notations were used to explain Y and functional parameters beta. Also, the Phi function used in equation (4) might be different from the equation above. A different notation for better explanations should be considered.

**Thank you for pointing out areas where the clarity of our mathematical explanations could improve. Please note that equation (4) is the same equation as equation (2), but written in a more compact (but standard) notation. We feel that it is useful to have both because it highlights the main structure of the model. However, we agree that the way that we worded it makes the link between Equation (2) and (4) obscure. To clarify, we have added the following text on line 107:**

**“Note that equation 4 is just a compact representation of equation 4.”**

**We also thank the reviewer for pointing out that we forgot to explicitly state that the Phi function must be the same in all these equations:**

**Line 107: “$\Phi$ links the covariate $X_{il}$ and the regression slopes $\beta$, so by using a generic $\beta$ in (4) we allow the conditional mean to be equal to any of the conditional means in the previous equations.”**

1.3 In Lines 263-265, L1 and L2 norms are mentioned in (iv). Although the results can be put into the supplementary material, a discussion about L1 and L2 norms is still meaningful in the main text.

**Indeed section (iv) on line 264 states: ‘whether L1 or L2 norms are used in FRECL when computing the residuals in an iteration, see equation (5); we generate 50 simulations for each of the scenarios with K = 3; these results are included in the supplementary material.’**

**We now include a definition of L1 and L2 norms in the main text, as well as a brief discussion about choosing one or the other:**

**Line 269: whether the L1 or L2 norms are used in FRECL, see equation (5), to quantify the 269 magnitude of the fitted residuals in an iteration. Recall that for a function f(s), 270 s ∈ T , its L1 norm is given by R T |f(s)|ds whereas its L2 norm is [R T (f(s))2ds] 1/2 . 271 We generate 50 simulations for each of the scenarios with K = 3; these results are 272 included S2 Fig. As the L1 and L2 norms performed nearly identically, we chose 273 to only use the L2 norm in the remainder of the manuscript.**

**Line 490: Selection of hyperparameters, such as the choice of using $L_1$ and $L_2$ norms may depend on the specific application and will need to be re-assessed when applying the method to new data sets.**

1.4 Related to the above comments, in the main text simulation results display, the L2 norm was automatically chosen for every sub-section. It would be better to state why only the L2 norm is used in other simulation comparisons.

**Thank you for this suggestion:**

**Line 273: As both $L_1$ and $L_2$ performed nearly identically, we chose to only use $L_2$ in the remainder of the manuscript.**

1.5 In the “Comparison with other methods” section, it will also be interesting to see the performance of other competing methods (considering observations formed with the values of both the explanatory and response variables) in the simulation study, not only based on response variable Y.

**The reviewer is absolutely correct that both comparisons are necessary for validating the method. We actually already provided this data in S2 Table, but we forgot to reference this in the main text. Our graphs only show the best-performing versions of the algorithms (using Y only) to improve clarity. We now reference it in the text:**

**Line 302: When we compare methods for the simulated data, we use $Y$ only in the main text, but the methods were also evaluated with $X$ and $Y$ appended together, which is shown in S2 Table.**

1.6 It is interesting to investigate the convergence properties of FRECL under the IID random error. A different (might be early) convergence rate might be expected.

**FRECL performed best under AR(1) which is why we chose to focus on this scenario when we modelled convergence (see Fig 3). One of the underlying assumptions of the FRECL method is that the K-means algorithm must converge. Our aim in the convergence results section was to experimentally verify that this property was met and also to highlight to the reader that they need to experimentally test convergence in their own dataset. These conclusions are already successfully presented in the manuscript, as is.**

**While we agree that there may be different convergence properties under different kinds of random error, we did not feel like this would influence the reader’s confidence in FRECL, nor influence FRECL’s usability. Therefore, we feel like this analysis– although potentially interesting– lies beyond the scope of this project.**

Minor comments

1.7 Line 54, the expectation of error should have brackets.

**Good catch– fixed.**

1.8 Lines 89 and 90, “our methods” and “each predictor” should be “our method” and “each predictor”.

**Typos now fixed.**

1.9 Line 119, you used a new notation, k ̂(i). An explanation or definition is expected in lines 123 and 124 for easier understanding.**This now reads:**

**Line 122: Reassign the $i$th observation, $i=1,\ldots,m$ to the best fitting model, which we refer to as $\hat k(i)$, a function that returns the assigned cluster designation for each observation: $\hat k(i) \in \{1,\ldots, K\}$, thus defining a new partition $P^+$**.

1.10 Line 208, the “5) in 20” expression for the threshold of filtering lowly expressed genes is confusing.

**This section was expanded for clarity:**

**Line 210: Some genes were lowly expressed in nearly all time points. To filter these out, we selected genes that were expressed at moderate levels in 20 or more time points in each season. We define moderate expression levels as those that surpass $5$ transcripts per million (TPM), which is a unit of gene expression after normalising RNA-seq data by the sequencing library depth and gene size. Using a TPM threshold of 5 is a common strategy used in biology for filtering out very lowly expressed genes \cite{Ezer2017}**

1.11 Line 212, “loess” is short for Locally estimated scatterplot smoothing, which should be capitalized. Also, the approach’s full name should be stated the first time it is used in the manuscript.

**LOESS is now capitalised everywhere and the full name is included on first mention.**

1.12 Figure 5 caption, please define or clarify the definition of “final ARI”.

**We have added the following text to the caption:** **final ARI after performing consensus clustering on the individual runs.**

1.13 The arrangement of Figure 7 could be improved to enhance clarity. It would be better to use A, B, C, and D letters for the top left, top right, bottom left, and bottom right to point out each sub-figure. The current layout is confusing.

**This has been implemented and we agree that it looks much better. The legend now reads:**

**On the basis of the elbow method, we selected K=10 clusters, both for the data set with 23 time points spread over one day (A); and for the data set with 24 time points spread over two days (B). The clusters are approximately evenly sized, under both conditions tested: over one day (C) or two days (D).**

Reviewer #2: The manuscript presents a significant contribution to the field of gene expression analysis through a novel clustering method. By addressing the comments and suggestions provided, the authors can enhance the clarity, readability, and overall impact of their work. The method's robustness, supported by comprehensive validation and practical application, underscores its potential for advancing biological insights in gene expression studies.

This manuscript presents a novel method for clustering gene expression data using a function-on-function regression model. The proposed method aims to identify clusters of genes with similar changes in their expression profiles under different experimental conditions, providing useful biological insights. The manuscript includes a detailed methodology, validation through simulations, and an application to real gene expression data.

It is a well-written manuscript with several strengths. Firstly, the manuscript introduces a novel clustering method that leverages functional regression models, allowing for multiple functional explanatory variables. This approach is innovative and addresses a gap in the current methods for clustering gene expression data. Secondly, the authors validate their method through extensive simulations, demonstrating its effectiveness compared to existing methods. This thorough validation adds significant credibility to their approach. Lastly, the application of the method to gene expression data reveals clusters enriched for biologically meaningful functions, highlighting the potential of the method to uncover novel biological insights.

2.0 Nevertheless, it could be better if the authors make any improvements with clarity in method description, comparison with alternative methods, and about real data application. While the methodology is described in detail, some sections could benefit from additional clarity and simplification to make the complex statistical methods more accessible to a broader audience. The manuscript compares the proposed method with several existing clustering methods. However, more detailed discussions on why certain methods perform better or worse in specific scenarios would be beneficial. The application to real gene expression data is compelling, but additional context on the biological implications of the findings could strengthen this section.

**Thank you for these positive comments. We believe that the general suggestions in the last paragraph are addressed in response to the specific queries below.**

Here are three questions for discussion.

2.1 Parameter Selection: How sensitive is the proposed method to the selection of parameters such as the number of clusters (K) and the choice of functional principal components? Can the authors provide guidelines or criteria for optimal parameter selection in practical applications?

**The impact of parameters are already discussed in the text, including the interaction of combinations of parameters. These are numbered in line 256. These include: (i) the assumed distribution of the random error term (ii) K (iii) sample size (iv) choice of L1 or L2 norms (v) number of iterations per run (vi) number of runs.**

**Regarding your comment on K, this corresponds to experiment (ii) above, already discussed in the paper.**

**Regarding FPCs, we do not use these in FRECL, though some of the alternative methods that we compare our approach to do.**

**We agree that we need to provide guidelines for parameter selection in our discussion. We have added the following text:**

**Line 493: However, our simulation results suggest the following guidelines: (i) A choice of L1 or L2 norm does not appear to have a large impact on the outcome. (ii) If possible, proceed with each run until convergence. (iii) Increase the number of runs until the consensus clustering converges. (iv) Select the number of clusters using a classical method, like the edge method.**

2.2 Computational Efficiency: Given the iterative nature of the proposed algorithm and the use of consensus clustering, what are the computational demands of the method? How does it scale with increasing data size, and are there any strategies to improve its efficiency?

**We agree that this is very important information to include in the Discussion.**

**Line 498: A downside of FRECL is that it is computationally intensive, specifically Algorithm 1. The runtime of Algorithm 1 of FRECL is a product of the number of runs, the number of iterations per run, $K$, and the runtime of the selected functional regression algorithm (which inherently depends on the size of the dataset). The number of iterations per run until convergence is not easily empirically calculated, although it can be experimentally determined for a specific data set, as we have shown. The number of iterations per run until convergence depends on $K$ and the size of the dataset. However, parallelisation is easily implemented, as each run can be computed on an independent node on a computing cluster.**

2.3 Biological Validation: The manuscript demonstrates that the identified clusters are enriched for genes with similar biological functions. Can the authors provide examples of specific biological pathways or processes that were identified using their method and discuss their significance?

**We have expanded this section to include recent work that highlights the significance of the biological pathways highlighted. This has greatly strengthened the manuscript.**

**Line 429: Interestingly, we find that ribosome and photosynthesis related genes tend to cluster together in FRECL, which may suggest that the same set of genes regulates the season-dependent gene expression of both processes, suggesting an avenue of research for biologists. Intriguingly, a recent manuscript highlights that both ribosomal processes and photosynthesis are downregulated during plant early age-related senescence, the process by which plants plan leaf death to enable energy expenditure in reproduction. As the seasons progress, we would expect that the plants will mature, so our results are consistent with these findings. In addition, this highlights a strength of our approach: normally ribosome and photosynthesis genes would not be clustered together due to having distinct temporal expression patterns, but we correctly identify that these two processes both change in the same way over the larger temporal scale.**
